# Emphysematous Gastritis: A Case Series on a Rare but Critical Gastrointestinal Condition

**DOI:** 10.7759/cureus.50409

**Published:** 2023-12-12

**Authors:** Abeer Qasim, Shalini Penikelapate, Franklin Sosa, Abhilasha Jyala, Haider Ghazanfar, Harish Patel, Anil Dev

**Affiliations:** 1 Internal Medicine, BronxCare Health System, New York, USA; 2 Internal Medicine, BronxCare Health System, Bronx, USA; 3 Internal Medicine, Bronx Lebanon Hospital Center, New York, USA; 4 Gastroenterology, BronxCare Health System, New York, USA

**Keywords:** necrotizing infection, conservative management, gas gangrene of stomach, gas-forming gastritis, pneumomatosis gastritis

## Abstract

Emphysematous gastritis (EG) is a rare and life-threatening condition characterized by gas-forming microorganisms causing gas to accumulate within the stomach wall. It has a high mortality rate and is associated with risk factors like gastroenteritis, alcohol use disorder, diabetes mellitus, renal failure, recent abdominal surgery, long-term corticosteroid use, and ingestion of corrosive agents. Diagnosis is challenging due to its rarity and nonspecific symptoms, including severe abdominal pain, coffee-ground emesis, fever, and signs of systemic infection. We present two cases of patients with signs and symptoms of EG, where prompt diagnosis and treatment were achieved, avoiding further complications. Surgical intervention was avoided due to the successful response to conservative treatment. These cases highlight the importance of early detection and intervention in improving patient outcomes and preventing complications associated with EG.

## Introduction

Emphysematous gastritis (EG) is an uncommon and life-threatening medical condition characterized by gas within the stomach wall. Fraenkel described the first documented case of EG in 1889 [1], and it is associated with a high mortality rate ranging from 55% to 61% [2,3]. Emphysematous gastritis represents a severe variant of gastritis primarily caused by gas-forming bacteria such as *Streptococcus* species, *Escherichia coli*, *Enterobacte*r species, *Clostridium* species, *Pseudomonas aeruginosa*, *Staphylococcus aureus*, *Candida* species, and *Mucor* species being prominent culprits [4]. In typical cases, a computed tomography scan reveals gas appearing non-linearly along the inner surface of the stomach wall [5]. Several EG risk factors have been identified, including gastroenteritis, alcohol use disorder, diabetes mellitus, end-stage renal disease, prolonged use of corticosteroids or nonsteroidal anti-inflammatory drugs (NSAIDs), previous abdominal surgery, and caustic ingestion [2]. Given its critical nature, EG necessitates immediate medical attention and management. Nevertheless, its rarity and the limited number of reported cases make it challenging for clinicians to diagnose and treat it effectively. Therefore, timely identification and appropriate intervention are essential to enhance patient outcomes and reduce the mortality associated with this condition.

## Case presentation

Case 1

**Figure 1:**
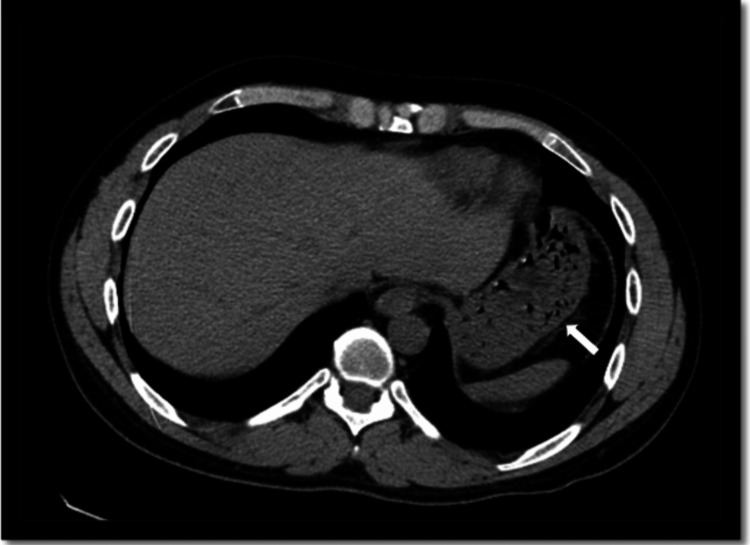
Axial view of CT abdomen and pelvis without contrast Circumferential thickening of the gastric wall with multiple small collections of air within the gastric wall and mild peri-gastric fat stranding (white arrow points to intramural gas in the stomach).

**Figure 2:**
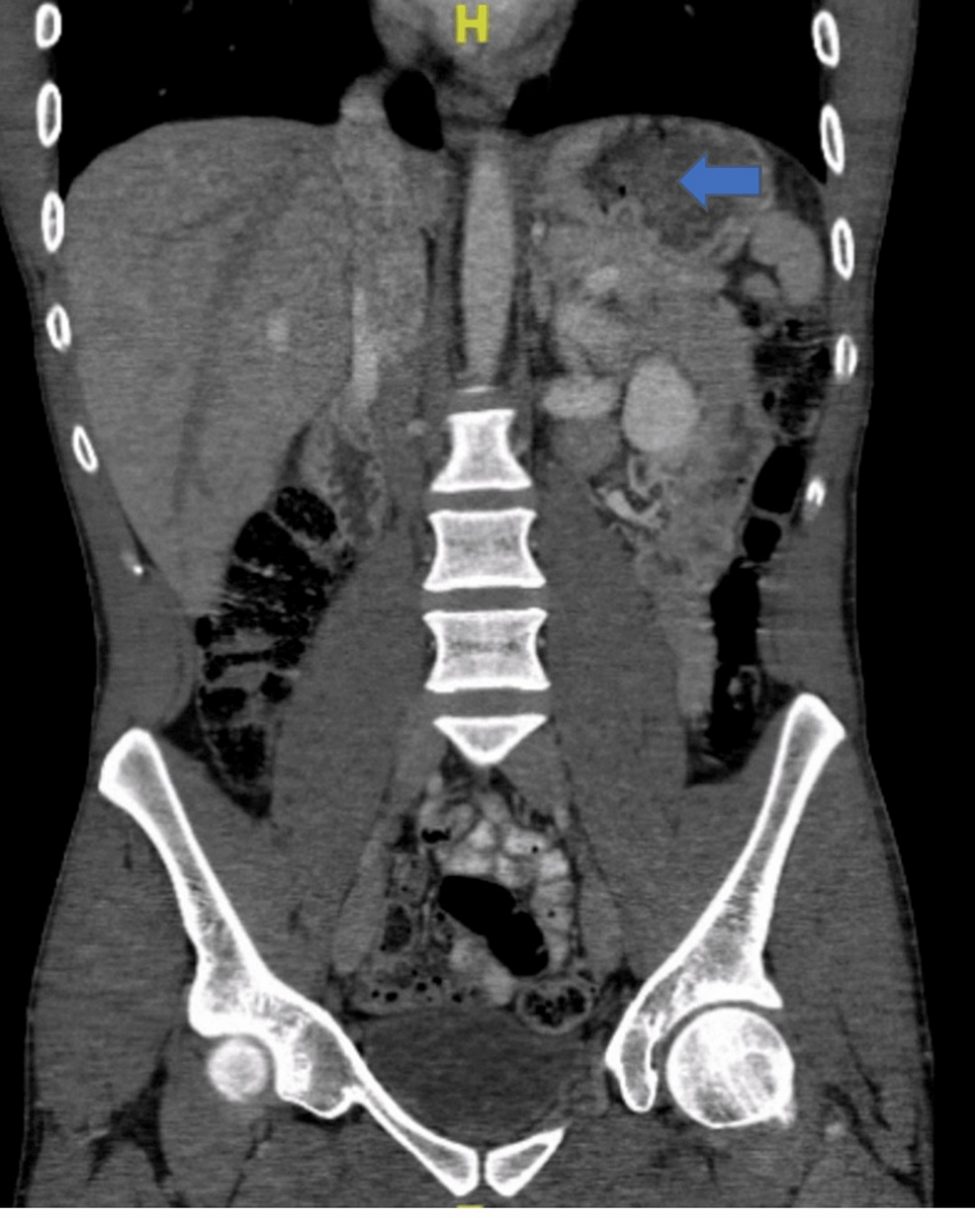
Coronal view of CT abdomen and pelvis without contrast Circumferential thickening of the gastric wall with multiple small collections of air within the gastric wall and mild peri-gastric fat stranding (blue arrow points to intramural gas in the stomach).

A 35-year-old man with a medical history of asthma and hearing loss due to a prior head injury presented to the emergency room with complaints of abdominal pain, non-bilious vomiting with specks of blood, and five episodes of diarrhea over the past two days. He also reported a fever with chills, a dry cough, and nasal congestion. The abdominal pain was diffuse but mainly localized to the lower and middle abdomen, described as sharp, and scored 7/10 in intensity with no radiation. His home medication included only albuterol (as needed), and he denied any use of NSAIDs. On physical examination, the patient was febrile with a temperature of 101.8°F, tachycardic with a heart rate of 125 beats per minute, and hypertensive with a blood pressure of 150/100 mmHg. Abdominal examination revealed tenderness in the middle and lower abdomen with guarding but preserved bowel sounds. Initial laboratory investigations showed a hemoglobin level of 14 g/dl and a leukocyte count of 11.7 x 10^9 cells/L, with a neutrophilic predominance of 79%. The patient had elevated liver enzymes, with aspartate aminotransferase (AST) and alanine transaminase (ALT) levels of 88/102 U/L and alkaline phosphatase of 114 U/L. His lipase level was mildly elevated at 56 U/L. A respiratory viral panel was positive for rhinoenterovirus and negative for COVID-19. The chest X-ray was unremarkable. The CT of the abdomen and pelvic imaging revealed circumferential thickening of the gastric wall with multiple small collections of air within the gastric wall and mild peri-gastric fat stranding, leading to the favored diagnosis of EG based on the clinical presentation and radiological findings (Figures 1-2). 

A nasogastric tube was placed for gastric decompression. He received IV fluids and once-daily IV pantoprazole, along with a combination of IV antibiotics (piperacillin-tazobactam, vancomycin, and metronidazole) and IV metoclopramide for antiemetic management. Blood cultures were negative. The patient's symptoms gradually improved during his hospital stay, and he tolerated oral feeding well. Follow-up laboratory tests showed improved liver enzyme levels and normalization of leukocyte counts. After completing five days of conservative management, the patient was discharged home.

Case 2

An 81-year-old man with a history of hypertension, dementia, chronic kidney disease, anemia, and benign prostatic hyperplasia with a chronic indwelling catheter was brought from a nursing home to the hospital with complaints of abdominal pain for three days. He reported one day of nausea and vomiting and an episode of coffee-ground emesis. A previous upper GI endoscopy one month ago revealed grade C esophagitis and chronic mild gastritis. The patient was recently admitted for abdominal pain and managed a large fecal ball with ileus, receiving an aggressive bowel regimen. His home medications include metoprolol tartrate, multivitamins, ascorbic acid, and ferrous sulfate; he denied any intake of corticosteroids or NSAIDs. On arrival, the patient had one more episode of coffee-ground emesis but remained hemodynamically stable. Physical examination revealed a distended, tympanic abdomen with rebound tenderness involving the left lower quadrant and normoactive bowel sounds.

**Figure 3:**
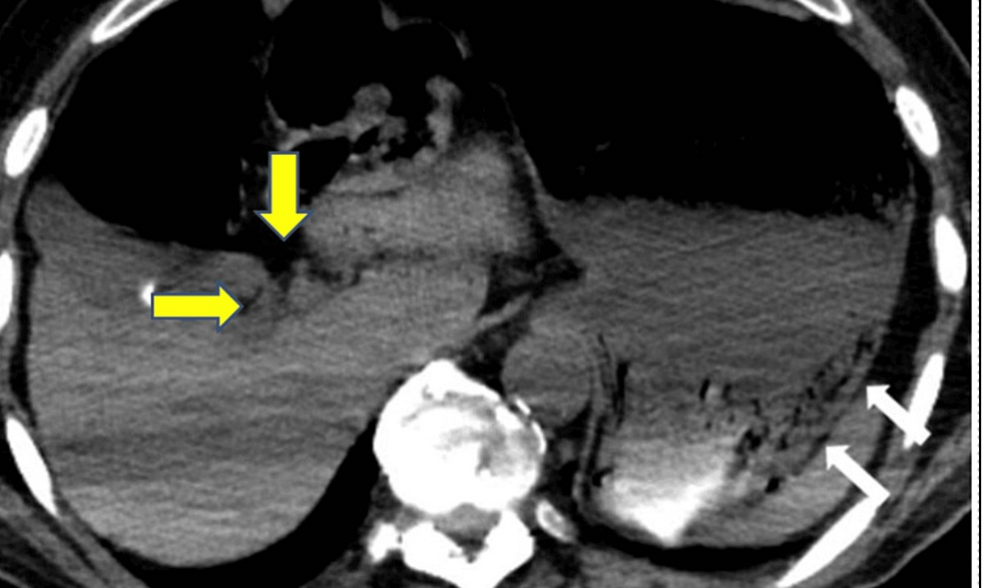
Axial view of CT abdomen and pelvis without contrast White arrows: Distended stomach with questionable pneumatosis within the wall of the fundus; Yellow arrows: Portal venous gas within the liver

**Figure 4:**
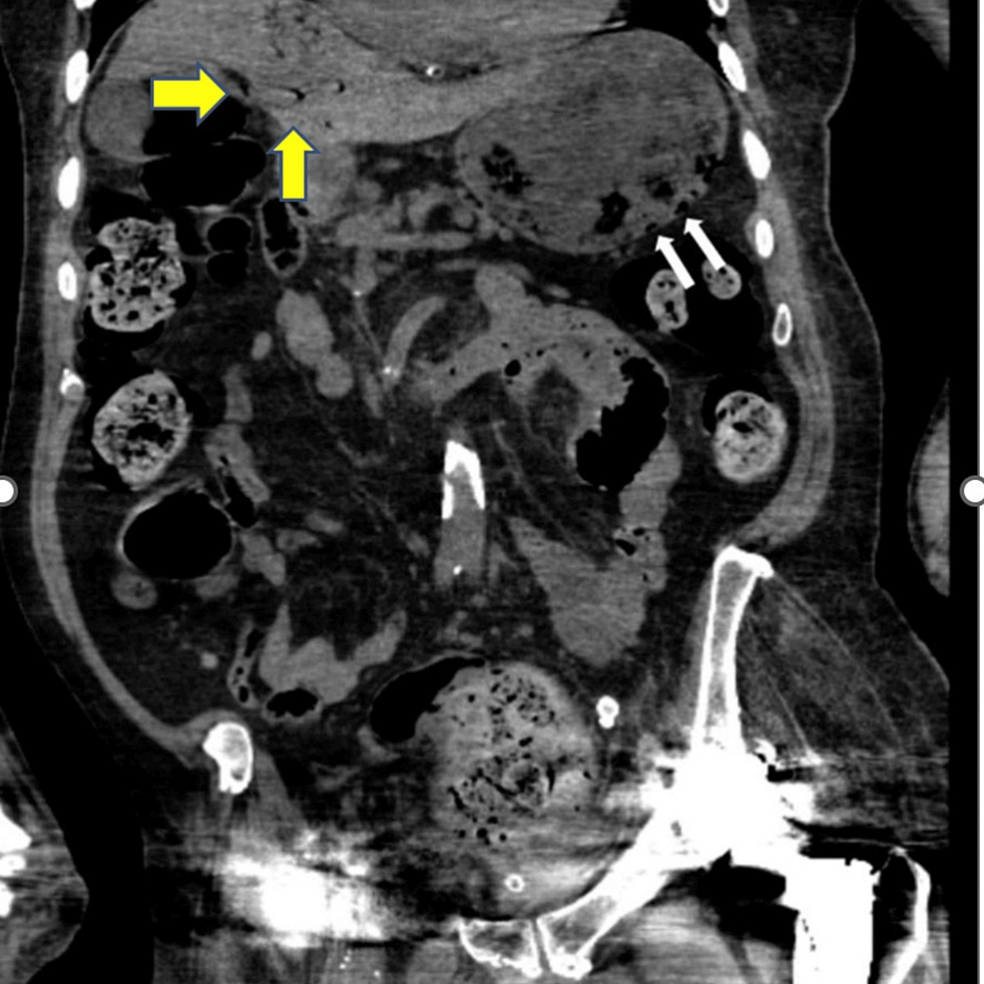
Coronal view of CT abdomen and pelvis without contrast White arrows: Distended stomach with questionable pneumatosis within the wall of the fundus; Yellow arrows: Portal venous gas within the liver

Initial investigations showed a hemoglobin level of 11.4 g/dl and a leukocyte count of 16.4 x 109 cells/L. Elevated lactic acid, blood urea nitrogen (BUN), and creatinine levels were noted (3.0 mmol/L, 72 mg/dl, and 1.6 mg/dl, respectively). Ammonia levels were elevated to 85 umol/L, while liver enzymes and bilirubin were within normal limits. The chest X-ray showed no acute cardiopulmonary abnormality. Non-contrast CT abdomen and pelvis demonstrated a distended stomach with questionable pneumatosis within the wall of the fundus, portal venous gas within the liver, and thickening of the wall of the ascending colon, suggestive of bowel ischemia and severe fecal impaction involving the rectum (Figures 3-4).

Emphysematous gastritis was favored due to his presentation and the radiological findings. The patient was admitted to the ICU, and a nasogastric tube was placed for decompression, revealing coffee-ground aspirate. Intravenous fluids and a combination of IV antibiotics (piperacillin-tazobactam, vancomycin, and metronidazole) were initiated. Gastroenterology was consulted for coffee-ground emesis and recommended a bolus of IV pantoprazole followed by twice-daily IV pantoprazole. The patient's constipation was managed with an aggressive bowel regimen involving enemas, lactulose, senna, docusate, and manual disimpaction. Follow-up labs showed normalization of ammonia levels. Urinalysis indicated pyuria, positive leukocyte esterase, bacteriuria, and crystals in the setting of a chronic indwelling catheter, but urine culture was negative and subsequently treated by replacing the Foley catheter. During his hospital stay, the patient showed clinical improvement, started oral feeds, and was transferred to the medicine floor. His symptoms resolved, and he tolerated feeding better. Follow-up labs revealed two sets of negative blood cultures and normalized labs to his baseline. The patient was discharged back to the nursing home on oral proton pump inhibitors after successfull conservative management.

## Discussion

**Figure 5:**
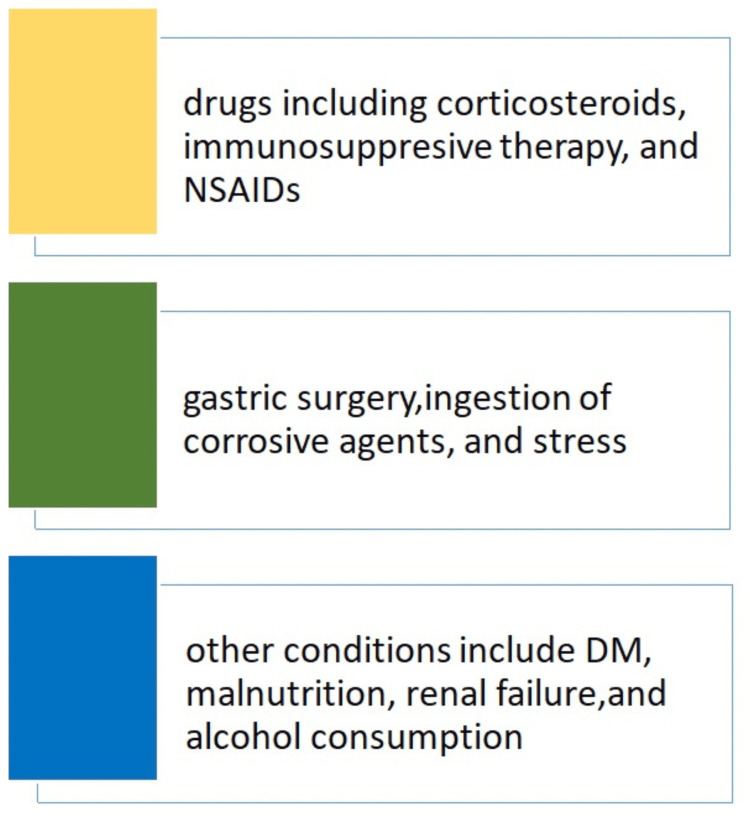
Risk factors associated with EG EG: Emphysematous gastritis, NSAIDs: Nonsteroidal anti-inflammatory drugs, DM: Diabetes mellitus Image created by author Qasim.

Emphysematous gastritis is a rare, life-threatening condition characterized by stomach inflammation and gas produced by microorganisms within the gastric wall [6]. It is a severe form of gastritis that can rapidly progress to necrosis, perforation, and sepsis. The clinicopathological description of this condition was initially documented by Fraenkel in 1889, and Weens first established its radiological diagnosis in 1946 [7]. It exhibits a striking clinical feature of abdominal pain, sepsis, and shock and is associated with a high mortality rate of 60% [8]. The most common causative organisms, including *Streptococci*, *E. coli*, *Enterobacter* species, *Clostridium welchii*, *S. aureus*, and* P. aeruginosa*, account for most cases. Other causative organisms, such as *Proteus* species and *Candida*, are also implicated [9]. *Mucor* species, a spore-forming anaerobic bacterium that ferments carbohydrates, producing gas and toxic substances, has been recognized as potential infectious agents associated with EG [10-11]. The risk factors associated with EG [12-13] are presented in Figure 5.

**Figure 6:**
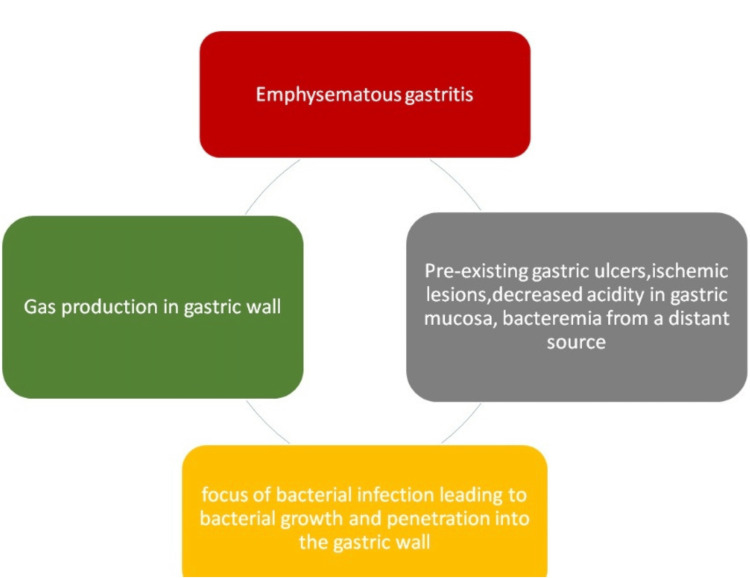
Pathogenesis of EG EG: Emphysematous gastritis Image created by author Qasim.

Numerous underlying factors that can lead to the damage of the gastric mucosal barrier have been associated with the development of this condition. The precise pathophysiology of EG remains uncertain; however, it is believed that pre-existing gastric ulcers or ischemic lesions create a favorable environment for bacterial infection, leading to their proliferation and infiltration into the gastric wall. Moreover, reduced acidity or the absence of lesions in the gastric mucosa might enable bacteria to colonize the stomach lining. Alternatively, the bacteria can spread through the bloodstream from a remote septic source, leading to EG [14,15]. The pathogenesis is presented in Figure 6.

The clinical presentation of EG can be nonspecific, making early diagnosis challenging. Common symptoms include severe abdominal pain, nausea, vomiting, hematemesis, and signs of systemic infection such as fever and tachycardia. The clinical signs in our first patient include abdominal pain, non-bilious vomiting with specks of blood, and five episodes of diarrhea. These symptoms were also associated with fever with chills, dry cough, and nasal congestion. Our second patient presented with more severe symptoms, including abdominal pain, nausea, vomiting, and an episode of coffee-ground emesis. Patients may appear critically ill with signs of sepsis, including hypotension and altered mental status [15]. Emphysematous gastritis is a critical medical condition that should be taken into account when evaluating acute abdominal cases, especially in the presence of risk factors. Timely diagnosis and intervention are essential, and urgent abdominal CT scanning is required to aid in early detection and management [16].

The definitive diagnosis of EG can be achieved through radiological evidence of gas within the stomach wall. The CT scan is the preferred imaging method, which shows the noticeable changes in the stomach, such as thickened folds in the inner lining of the stomach and swelling, as well as pockets of air trapped within the gastric wall. In some instances, air may also be observed in the veins that drain blood from the stomach and even in the portal vein [17]. Both of our patients underwent a CT scan immediately without any delay, which is crucial for optimizing patient outcomes in EG. If necrotic tissue is present, nasogastric intubation can provide valuable insights to aid the diagnosis. Moreover, the CT scan of the abdomen helps differentiate EG from other forms of acute abdomen. It can also be seen on the abdomen as linear, thin lucencies along the stomach wall [18]. Other imaging modalities, like ultrasound, may also aid in the diagnosis. Esophagogastroduodenoscopy (EGD) plays a crucial role in EG by assessing the extent of the condition, detecting signs of gastric tissue damage, and ruling out other potential gastrointestinal disorders [19].

The treatment of EG typically involves a combination of medical and supportive measures. It is a serious condition, and immediate medical attention is essential. The primary treatment approach involves initiating early antibiotic therapy that targets anaerobic bacteria and gram-negative Bacilli, along with administering IV fluids for hydration [20]. Both individuals in our study were started on a comprehensive range of antibiotics, a crucial step that aided us in ensuring timely and suitable intervention. This approach is vital for reducing the potential for these unfavorable consequences.

Adequate nutrition should also be initiated. Surgical management of EG is usually reserved for severe cases or when there are complications that cannot be adequately addressed with medical therapy alone. The decision for surgical intervention depends on the patient's overall condition, the extent of gastric necrosis, the presence of perforation or abscess formation, and the response to initial medical treatment [1]. It is crucial to tailor the treatment to each patient's unique situation, and the management should be done in close coordination with a team of healthcare professionals, including gastroenterologists, surgeons, infectious disease specialists, and critical care specialists. Early diagnosis and prompt, appropriate treatment are essential to improve the patient's outcome and reduce the risk of complications.

It is important to differentiate this condition from the other possible causes of gas-producing bacteria, which include gastric emphysema. It is crucial to distinguish between these two conditions as they exhibit distinct clinical symptoms, radiological features, treatment approaches, and prognosis. The differences are listed below in Table 1 [21]. Typically, when EG is detected early and immediate, proactive medical intervention is administered, such as prompt antibiotic therapy and comprehensive supportive care, the outlook is more positive. Effective infection control and addressing the root causes contribute to increased chances of patient recovery.

**Table 1:** Difference between EG and gastric emphysema EG: Emphysematous gastritis

Variables	Emphysematous gastritis	Gastric emphysema
Pathophysiology	Caused by gas-producing organisms including *Streptococci*,* E. coli*,* Enterobacter* species, *C. welchii*, *S. aureus*, and *P. aeruginosa *	A non-infectious origin of gas within the walls of the stomach.
Risk factors	Diabetes mellitus, prolonged use of corticosteroids, immunosuppression, and alcohol intake	Ingestion of gas-producing substances (e.g., carbonated beverages), mechanical disruption of the gastric wall (e.g., during endoscopy or surgery), or spontaneous release of gas from the surrounding tissues into the stomach wall, gastroenteritis, and gastric outlet obstruction
Symptoms	Common symptoms include severe abdominal pain, nausea, vomiting, hematemesis, and sepsis in severe cases	Gastric emphysema is typically asymptomatic
Radiological Findings	Computed tomography (CT) scans provide more detailed information, showing intramural gas and associated signs of gastric wall inflammation and thickening. Endoscopy can also aid in diagnosing emphysematous gastritis, allowing direct visualization of the gastric mucosa and gas bubbles within the stomach wall.	A CT scan shows hypodensities in the gastric wall
Treatment	Treatment typically involves aggressive supportive care, including fluid resuscitation and broad-spectrum antibiotics to target the gas-forming bacteria. Surgical intervention may be necessary in cases of gastric perforation, extensive necrosis, or signs of peritonitis.	Usually self-limiting
Prognosis	Bad prognosis with a high mortality rate	Good prognosis

## Conclusions

Emphysematous gastritis is an unusual but critical diagnosis to consider in patients with abdominal pain and suggestive radiological findings. Early identification and prompt initiation of appropriate treatment are vital in managing this potentially life-threatening condition. Advances in imaging technology, such as CT scans, have significantly improved our ability to diagnose this condition, leading to better outcomes. The importance of our case series highlights that timely and appropriate management is essential to minimize the risk of these adverse outcomes, especially as delayed diagnosis or inadequate treatment can result in rapid disease progression and increased morbidity and mortality. Further research and the accumulation of clinical evidence are necessary to enhance the recognition and treatment of this rare condition.
